# Activated c-SRC in ductal carcinoma *in situ* correlates with high tumour grade, high proliferation and HER2 positivity

**DOI:** 10.1038/sj.bjc.6603444

**Published:** 2006-10-24

**Authors:** G R Wilson, A Cramer, A Welman, F Knox, R Swindell, H Kawakatsu, R B Clarke, C Dive, N J Bundred

**Affiliations:** 1 Department of Academic Surgery, Research and Education Building 2nd floor, South Manchester University Hospital, Southmoor Road, Wythenshawe, Manchester M23 9LT, UK; 2 Department of Pathology, South Manchester University Hospital, Southmoor Road, Wythenshawe, Manchester M23 9LT, UK; 3 South Manchester University and Christie Hospital NHS Trusts, Manchester, UK; 4 Cellular & Molecular Pharmacology, Cancer Research-UK, Paterson Institute for Cancer Research, Manchester, UK; 5 Lung Biology Centre, University of California, San Fransisco, USA; 6 Breast Biology Group, Division of Cancer Studies, University of Manchester, Paterson Institute for Cancer Research, Manchester, UK

**Keywords:** c-Src, breast cancer, DCIS, immunohistochemistry, HER2

## Abstract

Overexpression and/or activity of c-Src non-receptor tyrosine kinase is associated with progression of several human epithelial cancers including breast cancer. c-Src activity in ‘pure’ ductal carcinoma *in situ* (DCIS) was measured to assess whether this predicts recurrence and/or correlates with HER2 expression and other clinical parameters. Activated c-Src levels were evaluated in DCIS biopsies from 129 women, with median follow-up at 60 months. High levels of activated c-Src correlated with HER2 positivity, high tumour grade, comedo necrosis and elevated epithelial proliferation. In univariate analysis, high activated c-Src level associated with lower recurrence-free survival at 5 years (*P*=0.011). Thus, high c-Src activity may identify a subset of DCIS with high risk of recurrence or progression to invasive cancer where therapeutics targeting c-Src may benefit this patient subset.

Overexpression and/or increased activity of c-Src non-receptor tyrosine kinase is associated with development of many human carcinomas including breast cancer (Irby and Yeatman, 2000) where it is elevated early in disease progression (Verbeek *et al*, 1996). c-Src overexpression is also associated with breast tumour metastasis (Guy *et al*, 1994; Muthuswamy and Muller, 1994), especially to bone (Myoui *et al*, 2003). The molecular events driving c-Src activation in breast cancers are unclear although this can occur downstream of activated receptor tyrosine kinases (RTKs) including HER2/erbB2 (Muthuswamy *et al*, 1994; Belsches-Jablonski *et al*, 2001). Overexpression of constitutively activated Src drives oncogenic processes that could contribute to latter stages of carcinogenesis and most prominently, increased motility and invasion (Frame, 2004). Few studies have examined subtypes of breast cancer with respect to c-Src activity and as Src-targeted therapies are entering the clinic, data are now required to determine which patient groups (disease type and stage) might optimally benefit.

There is a 12–20% recurrence rate at 10 years following breast conserving surgery and adjuvant radiotherapy for DCIS and up to half of recurrences are invasive carcinomas (Fisher *et al*, 1999). c-Src binds to, and can be activated by HER2 and appears to play a critical role in HER2-mediated breast cancer invasion and metastasis (Tan *et al*, 2005). Here, we investigated whether high levels of active c-Src are associated with clinico-pathological factors including HER2 status and early recurrence of DCIS.

## MATERIALS AND METHODS

### Patients and tissues

One hundred and twenty-nine cases of women diagnosed with ‘pure’ DCIS (median patient age 55 years, median follow-up 60 months) were selected from the University Hospital of South Manchester database. Thirty-five cases recurred within 5 years and 94 cases had not recurred. All specimens were formalin-fixed and paraffin-embedded. Patient age, histological nuclear grade, tumour size, follow-up and recurrence times were retrieved from our unit database (Barnes *et al*, 2005). Ethical approval was from South Manchester University Hospital Ethics Board.

### Immunohistochemistry and Western blotting

mAb Clone 28 recognizes an epitope adjacent to Tyrosine 530 in the C terminal regulatory domain of activated c-Src (Kawakatsu *et al*, 1996). Sections (4 *μ*m) of tissue were processed as previously described (Boland *et al*, 2004) before incubation with Clone 28 (1 : 800) for 60 min. Antibody binding was detected using anti-mouse DakoCytomation Envision+System HRP kit (Dako, UK). Incubation time with 3.3′-diaminobenzidine and substrate-chromogen depended on positive control staining intensity (∼8 min). Sections were counterstained as previously described (Boland *et al*, 2004). Mouse IgG2a*κ* (1 : 50) provided a negative control. HCT116 cells engineered to inducibly express wild-type (wt) or constitutively activated c-Src (c-SrcY527F) (Welman *et al*, 2005) were used as positive control pellets, processed and analysed identically to clinical specimens. Induction of wt c-Src (or c-SrcY527F) was confirmed by Western blotting using pAb rabbit anti-Src (pY418, Biosource, UK no. 44-660). Activated c-Src staining was also investigated in a MDA MB231 breast cancer cell tumour xenograft, chosen as this cell line exhibits high levels of activated c-Src measured by Western blotting (Ito *et al*, 2002). Activated c-Src expression was also evaluated in sections of normal breast ductal epithelium by IHC. Inter-assay variation was assessed in high and low scoring sections form one batch in the subsequent batches. All slides were examined and scored by two independent observers, one blinded to clinical outcome. Total cellular activated c-Src staining intensity was scored using a scale of 0 (negative), 1 (low), 2 (moderate) and 3 (high). To qualify as 3, 2 and 1 dark staining of 100, >70 or <70% of tumour cells had to be observed. The IHC evaluation of ER status, HER2 expression and Ki67 levels was as previously described (Boland *et al*, 2004). To assess intra- and inter-observer variability, 60 sections were selected at random and scored twice by the same observer (G.W., 4 of 60 scores differed) and scored independently by two observers (G.W. and A.C, 3 of 60 cases scores differed). Slides that differed were re-analysed in conference and a representative score agreed.

## RESULTS

### Activated c-Src staining in different breast tissue

**Figure 1:**
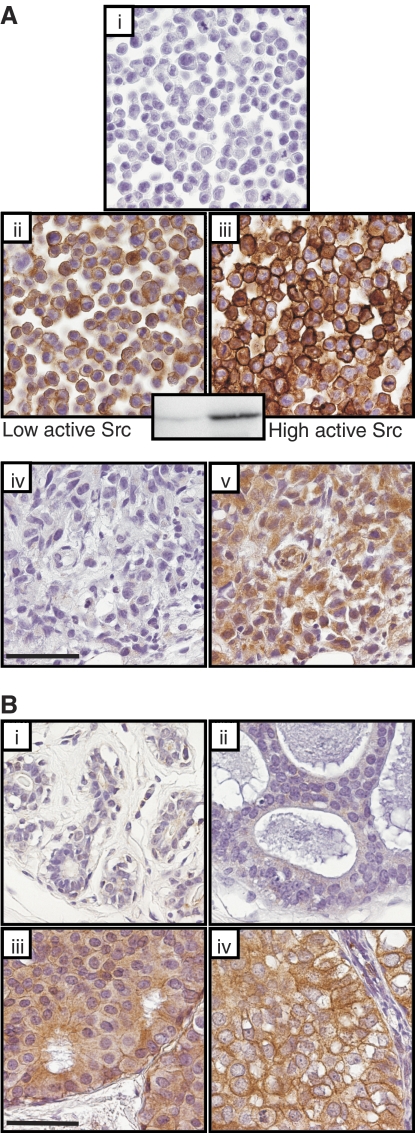
(**Ai–v**) Experimental controls for Clone 28 immunohistochemistry of activated c-Src. Pellets of untreated or doxycycline treated HCT116 colon cancer cells that express a doxycycline-inducible, wild-type (wt) c-Src. High levels of activated c-Src were detected in doxycyline treated HCT116 colon cancer cells harbouring the doxycycline-inducible wt c-Src (**Aiii**). Detectable but lower levels of active c-Src were detected in these HCT116 cells in the absence of doxycyline treatment (**Aii**). Induction of wt c-Src was independently confirmed by western blotting using an anti Src[pY^418^] antibody specific for autophosphorylated active c-Src (Figure 1A insert. No significant immunoreactivity was detected in HCT116 cells analysed by mouse IgG2a, which served as a negative control (**Ai**). Figure 1A(iv) shows a section of a MDA MB231 tumour xenograft negative control (IgG2A) and 1A(**v**) shows clone positive staining with Clone 28 in this xenograft indicative of high levels of activated c-Src. (**Bi–iv**) Immunohistochemistry for activated c-Src using clone 28 in normal breast epithelium (**Bi**), and DCIS tumours (**Bii–iv**). Low levels of activated c-Src in **B**(**ii**), moderate levels in **B**(**iii**) and high levels in **B**(**iv**). A higher level of activated c-Src was detected in DCIS tumours compared with normal breast. There was a positive correlation between the level of activated c-Src and tumour nuclear grade (*P*<0.0005). The scale bar equals 50 *μ*m.

Initially, the ability of clone 28 to recognize activated c-Src using the IHC protocols above was confirmed using HCT116 cell pellet controls. When high levels of wt c-Src (Figure 1A(iii), (or c-Src Y527F, not shown), were induced, staining was strongly positive compared to uninduced cells (Figure 1Aii). Controls with mouse IgG did not stain (Figure 1A(i)). Western blotting confirmed that wt c-Src was activated in HCT116 when induced (Figure 1 inset) thus these data together confirmed that clone 28 can reliably probe active c-Src by IHC. Figure 1A(v) shows positive staining with clone 28 staining of the MDA MB231 breast cancer cell xenograft consistent with the reported high levels of this breast cell line (Ito *et al*, 2002).

Activated c-Src staining was detected in all DCIS specimens with higher levels in DCIS compared with normal breast tissue, and correlated positively with tumour nuclear grade (Figure 1B(i–iv)). Low-level-activated c-Src was detected in 12% normal breast tissue sections. Of 129 DCIS tumours analysed 19% tumours expressed low activated c-Src levels, 41% expressed moderate and 40% expressed high levels. Moderate immuno-reactivity was often observed in lymphocytes surrounding DCIS.

### Activated c-Src expression *vs* clinico-pathological variables

Table 1 summarises the relationship between expression of total cellular activated c-Src and clinico-pathological factors. High levels of activated c-Src in DCIS was associated with HER2 positivity (*P*<0.0005), high tumour nuclear grade (*P*<0.0005), the presence of comedo necrosis (*P*=0.001) and high Ki67 scores (*P*=0.025), but not with ER status (*P*=0.973), tumour size (*P*=0.403) and EGFR/HER1 expression (*P*=0.507).

### Activated c-Src and recurrence-free survival

#### Univariate analysis

**Figure 2:**
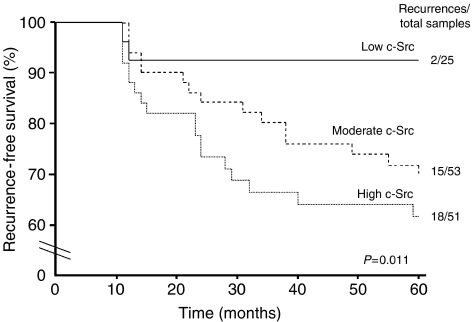
In univariate analysis, Kaplain–Meier plot of cumulative local recurrence (DCIS and invasive breast carcinoma) for 129 patients with DCIS. Low-activated c-Src expressing DCIS tumours had a significant disease-free survival advantage over DCIS expressing moderate to high levels of activated c-Src.

Activated c-Src level was a significant predictor of DCIS recurrence at 5 years (*P*=0.011). Patients with DCIS expressing higher levels of activated c-Src had a poorer cumulative 5 year disease-free survival compared to those with low levels (61.7 *vs* 92%, *P*=0.011, Figure 2). Of the 35 DCIS tumours that recurred by 5 years, 6% expressed low levels of activated c-Src, 43% expressed moderate levels and 51% expressed high levels (Figure 2). Other factors including HER2 status, Ki67, tumour nuclear grade, margin status and patient age were also significant predictors of disease recurrence (Table 2).

#### Multivariate analysis

Higher tumour nuclear grade, involved margins (<1 mm) and higher patient age (⩾50 years) were independently associated with an increased risk of recurrence at 5 years (Table 3). Activated c-Src level was not a significant independent predictor of recurrence-free survival.

### Activated c-Src *vs* recurrence pathology

Of the 35 women with recurrent DCIS, 37% had invasive recurrences and 63% had DCIS recurrence. The median recurrence time for invasive carcinoma was 24 months, compared with 13 months for DCIS recurrences. There was no significant difference between the level of activated c-Src and onset of DCIS recurrences (score low c-Src, 11 months; moderate, 12 months; high, 11 months). Of the 13 primary DCIS tumours that recurred as invasive carcinomas, 46% were moderate and 54% were high scores for activated c-Src. Of the 22 primary tumours that recurred as DCIS, 9% scored low 41% scored moderate and 50% scored high for activated c-Src.

## DISCUSSION

Available data on activated c-Src levels in breast cancer relates mainly to invasive breast carcinoma. Our data combined with that of others (Ito *et al*, 2002; Diaz *et al*, 2006; Madan *et al*, 2006; Planas-Silva *et al*, 2006), suggests that compared to normal breast epithelium, increased levels of activated c-Src are present in DCIS and invasive breast carcinoma. Activated c-Src levels correlated with HER2 expression, a higher tumour nuclear grade, the presence of comedo necrosis, and higher epithelial proliferative status. Furthermore, high expression of activated c-Src correlated with significantly lower recurrence-free survival at 5 years. Consistent with the physical interactions between HER2 and c-Src, we observed a significant association between HER2 expression and activated c-Src. These data also resonate with function testing studies where overexpression of HER2 in transgenic mouse systems (Muthuswamy *et al*, 1994) and mammary epithelial cells (Sheffield, 1998) that resulted in c-Src activation. HER2 activation in human breast cancer cells increased c-Src protein level by driving its synthesis and/or stabilisation (Tan *et al*, 2005). However, highly expressed activated c-Src was also seen in HER2 negative DCIS thus HER2 independent pathways for activation must exist.

Although HER2 can activate c-Src, it is unclear if c-Src promotes proliferation. Discrepancies exist between activated c-Src levels and proliferation in invasive breast carcinoma (Ito *et al*, 2002). Here, activated c-Src levels positively correlated with increased cell proliferation but we cannot ascribe causality and there was no significant correlation between activated c-Src and tumour size.

Although numerous studies have demonstrated elevated c-Src activity in breast carcinoma (Jacobs and Rubsamen, 1983; Ottenhoff-Kalff *et al*, 1992; Verbeek *et al*, 1996), there have been discrepancies between tumour histological grade and activated c-Src (Ito *et al*, 2002). Here, levels of activated c-Src in DCIS strongly correlated with tumour nuclear grade (Figure 1B (ii–iv)) and comedo necrosis.

Elevated c-Src activity leads to inhibition of matrix metalloproteinases (Noritake *et al*, 1999), disruption of cell–cell adhesions (Yeatman, 2004) and enhances the migratory potential of tumour cells (Frame, 2002). We report here, that moderate to high levels of activated c-Src in DCIS recurred as invasive breast carcinomas and that increased activated c-Src level was a significant, but not independent predictor of disease recurrence.

In conclusion, we detected elevated activated c-Src in HER2 positive, high nuclear grade, DCIS tumours with a higher proliferative index and comedo necrosis. Our data support the hypothesis that in DCIS, c-Src may play a role in tumour proliferation, development of higher grade lesions and may facilitate progression to invasive recurrences and as such represent tractable drug targets in both DCIS and invasive breast carcinomas. HER2 overexpression in invasive breast cancer is associated with an aggressive phenotype, resistance to hormonal therapy and poor survival (e.g. Slamon *et al*, 1987). Only a third of HER2-overpressing metastatic breast cancers respond to Trastuzumab (Herceptin), a function blocking anti-HER2 antibody (Vogel *et al*, 2002). c-Src targeted drugs are entering early clinical trials, and it is possible in appropriate patient cohorts that c-Src targeted drugs will be of benefit and that combination therapy with a c-Src inhibitor plus Herceptin may be superior to Herceptin alone.

## Figures and Tables

**Table 1:** Correlations between the total cellular expression levels of activated c-Src (low, moderate, and high) and clinicopathologic factors in 129 DCIS tumours

	**Level of activated c-Src**			
**Clinical Factor**	**Low (*n*=25)**	**Moderate (*n*=53)**	**High (*n*=51)**	***P*-value**
*HER1 status (n*=*43*)				
Positive (⩾2)	5	10	15	
Negative (<2)	3	6	4	0.507^a^
				
*HER2 status (n*=*125*)				
Positive (⩾2)	10	29	42	
Negative (<2)	15	21	8	<0.0005^a^
				
*ER status (n*=*126*)				
Positive	16	34	32	
Negative	8	18	18	0.973^a^
				
*Ki67 (n*=*125*)				
Median	11.2	11.0	13.7	
Range	2.5–31.4	1.3–61.1	2.6–46.0	0.025^b^
				
*Tumour nuclear grade (n*=*128*)				
Low	5	4	0	
Intermediate	11	21	6	
High	9	27	45	<0.0005^a^
				
*Histological type (n*=*125*)				
Comedo	3	11	11	
Mixed comedo	6	22	32	
Non-comedo	14	20	6	0.001^a^
				
*Tumour size (mm) (n*=*123*)				
Median	17.5	13.5	20	
Range	5–40	4–43	4–75	0.403^b^

High expression levels of activated c-Src in DCIS correlated with HER2 positivity, high tumour grade, the presence of comedo necrosis and higher epithelial proliferative status but not with tumour size, ER status and EGFR expression.

Statistical analysis:

a *χ*^2^ test.

b Kruskall–Wallis test.

**Table 2:** Univariate analysis of predictors of DCIS recurrence at 5 years using the log rank test

**Clinical factor**	**Non-recurrent (*n*=94)**	**Recurrent (*n*=35)**	***P*-value**
*HER2 status* (%) (*n*=*125*)			
Positive (⩾2)	52 (57.8)	29 (82.9)	
Negative (<2)	38 (42.2)	6 (17.1)	0.01
			
*Ki67 score* (%) (*n*=*125*)			
<12.2 (median)	52 (57.8)	10 (28.6)	
⩾12.2	38 (42.2)	25 (71.4)	0.006
			
*Activated c-Src* (%) (*n*=*129*)			
Low	23 (24.5)	2 (5.7)	
Moderate	38 (40.4)	15 (42.9)	
High	33 (35.1)	18 (51.4)	0.011
			
*ER status* (%) (*n*=*126*)			
Positive	61 (67.0)	21 (60.0)	
Negative	30 (33.0)	14 (40.0)	0.524
			
*Tumour grade* (%) (*n*=*128*)			
Low	9 (9.7)	0 (0.0)	
Intermediate	34 (36.5)	4 (11.4)	
High	50 (53.8)	31 (88.6)	0.0003
			
*Margins* (%) (*n*=*129*)			
Involved (<1 mm)	78 (83.0)	19 (54.3)	
Clear (⩾1 mm)	16 (17.0)	16 (45.7)	0.001
			
*Patient age (years) (n*=*129*) (%)			
<50	6 (6.4)	8 (22.9)	
⩾50	88 (93.6)	27 (77.1)	0.019

HER2 positivity, high epithelial proliferation, high levels of total cellular activated c-Src, high tumour nuclear grade, involved margins and young age at presentation were associated with a higher disease recurrence at 5 years.

**Table 3:** Independent predictors of DCIS recurrence in the patient group (*n*=129), carried out using Cox proportional hazards regression analysis

**Clinical factor**	**B**	**s.e.**	**Exp (B)**	**95% CI for Exp (B)**	***P*-value**
*Margin status*					
Involved (<1 mm)	0.967	0.344	2.63	1.34–5.17	0.005
					
*Patient age*					
<50 years	1.53	0.414	4.64	2.06–10.46	<0.0005
					
*Tumour nuclear grade*					
High	1.751	0.536	5.76	2.01–16.47	0.001
